# Ageing, Leisure, and Social Connectedness: How could Leisure Help Reduce Social Isolation of Older People?

**DOI:** 10.1007/s11205-012-0097-6

**Published:** 2012-06-13

**Authors:** Vera Toepoel

**Affiliations:** Department of Leisure Studies, Faculty of Social and Behavioural Sciences, Tilburg University, P.O. Box 90153, 5000 LE Tilburg, The Netherlands

**Keywords:** Social connectedness, Social support, Older adults, Social isolation, Intervention, Social indicators

## Abstract

This study investigates the relation between leisure activities and the social status of the elderly based on a heterogeneous sample of the Dutch population. Close relationships are also analyzed to identify which people could serve as successful stimulators of leisure participation. The social profile confirms that older people have fewer social contacts and often feel lonely. This study shows that leisure activities explain a significant part of older people’s social connectedness. Voluntary work, cultural activities, holiday, sports, reading books, hobbies and shopping are found to be successful predictors for social connectedness of older people. Watching TV, listening to the radio, and spending time behind the computer (passive activities) were not associated with social connectedness. Friends correlate positively to participation in leisure activities. Partners play a role in participation in cultural activities and sports; parents play a role in participation in voluntary work and holidays; siblings play a role in voluntary work and sports; and children play a role in cultural activities, reading books, and shopping. Local communities can use these close relationships and develop special programs to increase social connectedness and hence improve quality of life for older adults.

## Introduction

Much has been written about the relationship between ageing and social isolation. Work on ageing has been moving away from a social integration approach focusing on roles and activities toward a more network-orientated approach (Cornwell et al. 2008). Leisure can be an important tool in increasing or maintaining social integration in later life. Although social relations are a fundamental part of many leisure activities, especially for older adults, acquiring social contacts can be a primary goal. However, little is known about how different forms of leisure activities can contribute to social integration or how social relationships contribute to participation in leisure activities.

Age has been found to be negatively related to network size, closeness to network members, and the number of non-primary-group ties (Cornwell et al. 2008). In addition, age is arguably positively related to the experience of loneliness (Bowling and Gabriel 2004; Giummarra et al. 2007). The consequences of social isolation include bad health, depression, personal disorders, and suicide (Findlay 2003; Pettigrew 2007). Given the association between ageing and loss of social contacts, these negative consequences make the prevention and treatment of social isolation an important priority in ageing populations.

The primary research objectives of this study were to investigate the relationship between social isolation and leisure activities, in order to suggest potential consumption-related strategies that may be effective in improving quality of life for older adults. A profile is developed using data from the LISS Panel, a representative sample of the Dutch population, consisting of more than 8,000 individuals aged 18 and older. Five dimensions of social connectedness (number of social gatherings, number of close relationships, satisfaction with social contacts, feeling of social connectedness, and loneliness) were investigated in relation to leisure activities. In addition, the relationship between type of social relationships and leisure participation was analyzed. Comparisons were made between the adult population and older age groups. Although these data come from Dutch respondents, there is no reason to assume that results cannot be generalized to other populations.

## Background

Many countries now face a major change in the age-structure due to falling birth-rates and the ageing of the baby boom-generation. Increasing proportions of older people go along with declining proportions of younger people. The growth in the number of older people has lead to an increase in public expenditures for this group due to higher medical costs and old age pensions. Governments are looking for ways to reduce these costs. One of the possibilities to reduce medical costs seems to be the enhancement of quality of life in older age (Bowling and Gabriel 2004). Most public policy debates are concerned with the physical issues of ageing, while social issues such as isolation tend to be ignored. Social isolation is linked to poor physical and mental health, however (Findlay 2003; Pettigrew 2007). Therefore, the prevention of social isolation seems to be an important step in increasing the quality of life of older people and reducing public expenditure on medical costs for this group.

‘Social isolation’, ‘social integration’, ‘social capital’, and ‘social connectedness’ are just a few of the terms used to describe concepts related to sociability in societies. All of these are contested terms, which often result in intense debates about what is meant when the terms are used (Jeannotte 2008). In addition, they are also notoriously difficult concepts to operationalize, and there does not seem to be any consensus on how to measure them. Jenson (1998) identifies five dimensions of social integration, one of them being isolation. Social isolation can therefore be seen as an indicator of social integration. Social capital, at least in the resource-based approach^1^ of Bourdieu (1984, 1986) and Coleman (1988), often refers to the quality and quantity of social relations that may provide individuals with access to resources (Portes 1998). When people are socially isolated, they have limited social relationships and as a result limited access to resources. This paper follows De Jong Gierveld and Van Tilburg (2006) in using the term “social isolation” when referring to the lack of social ties of older people; the term “social integration” is used to refer to the opposite of social isolation; and “social connectedness” is used when referring to the amount and quality of social relationships of older people [see Bourdieu (1984, 1986) and Coleman (1988)].

### Social Isolation and Ageing

Social disengagement theory argues that ageing can be thought of as a mutual withdrawal or disengagement which inevitably takes place between the ageing person and others. The process leads to a relinquishment of roles since the ageing person drops out of the working sphere and children move out of the house. In addition, older people face a reduction of ties since peers start to die off. This process is conceived as removing the individual from a certain amount of normative control, free to become more individualized and less likely to be easily assimilated into new groupings (Cumming and Henri 1961).

Giummarra et al. (2007) interviewed health professionals and older people to shed more light on the relation between ageing and social isolation. Older people found social connectedness and social activity to be strongly associated with overall health. They described social and mental health as being even more important than physical health. In particular, older people associated loneliness with poorer overall health. In addition, Giummarra et al. note that health professionals reported mental and physical health deteriorating when older people are socially isolated. They found social health to be at least as important as mental and physical health. “Social health is dependent on social connectedness as well as the extent to which communities value diversity, are supportive and inclusive, and provide opportunities for each person to participate in community life, as well as the number and quality of social supports and relationships that a person maintains” (Giummarra et al. 2007, p. 643).

Social integration is essential to successful aging (Cornwell et al. 2008) because it provides embeddedness in systems of norms, control, and trust, access to information and other resources, as well as social support (see, e.g. Bourdieu 1986; Coleman 1988; Putnam 1995). Having many ties to other people gives people alternative routes to valuable resources such as information, social support, financial connectedness, or cultural connectedness through connections to experts (Coleman 1988). Such resources are essential for well-being and can serve to improve quality of life (Cornwell et al. 2008; Hause et al. 1988). Bowling and Gabriel (2004) found measures of social isolation (i.e., social activities, social support, and feelings of loneliness) to be important indicators for quality of life. These measures were found to be more important than socio-demographic and socio-economic indicators in an integrated model of quality of life. Interaction with strong ties (Lin et al. 1985), having a confidant (Grenade and Boldy 2008), and family (Fiori et al. 2006) were found to be most beneficial.

### Leisure and Ageing

Pettigrew (2007) argues that individuals can exert some degree of control over their experience of loneliness. Leisure activities are often perceived to be instrumental in determining whether increasing levels of social isolation experienced with advancing age result in feelings of loneliness. Continuity theory is often used in the study of older people’s leisure behavior (see e.g. Burnett-Wolle and Godbey 2007; Godbey 1994; Hicks 1997; Mannel and Kleiber 1997). Continuity theory holds that, in making adaptive choices, middle-aged and older adults attempt to preserve and maintain existing internal and external structures and prefer to accomplish this objective by using strategies tied to their past experiences and their social world (Atchley 1989). Continuity theory assumes that older people who maintain outgoing leisure behavior (e.g. going out and seeing other people as they age) have higher rates of well-being. This is demonstrated in a number of studies (see, a.o. Fernandez-Ballesteros et al. 2001; Longino and Cart 1982; Reitzes et al. 1995).

Many leisure activities have social outcomes either as the main goal (e.g., visits to friends or relatives) or as a byproduct of some other goal (e.g., attending a concert). Having a drink in a bar, visiting a festival, or going to the theatre, are occasions where the company of others is enjoyed and relationships with friends, relatives, and acquaintances are strengthened (Van Ingen and Van Eijck 2009). Pettigrew (2007) argues that eating and drinking rituals, reading, gardening, and shopping assist older people to remain socially active and act as a means of alleviating loneliness. Other leisure activities found to reduce social isolation include volunteering (Cornwell et al. 2008), cultural activities (Bourdieu 1984; Kraaykamp 2002), sports (Liu 2009; Scheerder et al. 2004), and the use of Internet (Firth and Mellor 2007). In contrast, Putnam (1995) found television viewing to increase social isolation. He states that television viewing impedes participation outside the home and takes the place of outside activities, social gatherings, and conversations. He argues that more television viewing implies less social trust, less membership in groups, and less social connections. What is unclear, however, is whether television viewing *contributes to* isolation or if it is a *consequence of* isolation.

### Leisure and Social Connectedness

Social connectedness, the quality and quantity of social relationships in networks, and its relationship with leisure has received a lot of attention in the leisure field (Arai 2000; Arai and Pedlar 2003; Blackshaw and Long 2005; Glover 2004a, b; Glover and Hemingway 2005; Warde and Tampubolon 2002). One important comment on the social connectedness literature in general is the circular logic of the concept. Social connectedness is simultaneously a cause and an effect (Portes 1998). It leads to positive outcomes, such as leisure participation, and its existence is inferred from the same outcomes. Having social ties stimulates an individual to do certain activities, because it facilitates social support (Van Ingen and Van Eijck 2009). Doing activities on the other hand results in social ties because of the social interaction involved in the activities (Longino and Cart 1982). Which mechanisms cause the virtuous circles within social connectedness or how effects arise as a result of social connectedness is unclear (Van Ingen and Van Eijck 2009).

Putnam (2000) argues that the type of leisure activity is crucial in generating social connectedness. He distinguishes between *doing* things together or productive activities, and *watching* together or consumptive activities. Productive activities are the ones which are active and creative, involve cooperation and are better for social connectedness while consumptive activities are the ones which involve just watching or experiencing. According to Putnam (2000), productive activities create stronger ties than consumptive activities.

Van Ingen and van Eijck (2009) state that besides the type of activity, the type of company is an important factor in evaluating the relationship between social connectedness and leisure. They argue that bonds with household members can facilitate social support, whereas bonds with wider social circles can stimulate civic engagement. Kyle and Cick (2002), in their study on the social nature of leisure involvement, found that the relationships people share with significant family and friends are the most important contextual element of leisure involvement. For the people involved, social contacts were both the focus of their involvement in leisure activities as well as the agent that maintained their involvement. Burch (2009) states that the (nature of) intimate social ties that surround an individual are crucial determinants of variation in leisure behavior. He argues that transactions with and socialization by one’s work mates, parents, spouses, and friends will shape the nature of one’s leisure behavior. Orsega-Smith et al. (2007) discuss the importance of social support for older adults in that having active friends or being encouraged by at least one person were the most influential stimuli to participate in activities. They argue that if an older adult is exposed to a leisure activity and participates with supportive friends, he/she will be more committed participating in that activity than someone who has no friends with whom to do those activities.

Stalker (2008) and Orsega-Smith et al. (2007), among others, argue that the character of social engagement among the elderly needs to be more fully accounted for by demonstrating what patterns of social behavior and network characteristics improve wellbeing in later life. As suggested by Grenade and Boldy (2008), there is a need for strategic thinking in order to provide older people with maximum opportunities to maintain a good quality of life. Therefore, this paper will analyze the social profile of older people based on a large sample from the general population including non-institutionalized older people. The role of different types of leisure activities on generating social connectedness is taken into account, as well as the role of different types of social relationships on leisure participation (virtuous circle). It is hypothesized that social connectedness decreases with age. In addition, it is hypothesized that participation in different types of leisure activities is related to greater levels of social connectedness, except for passive activities such as watching television. Close contacts are expected to influence participation in leisure activities.

## Design and Implementation

### Data Collection

Data come from an existing survey that was run in the LISS Panel (see www.lissdata.nl for recruitment and composition of the panel; the data used are publicly available at this website), an online household panel administered by CentERdata, which started in 2007. The panel is representative of the Dutch (speaking) population in the Netherlands, aged 16 years and over. Although the LISS Panel is an Internet-based panel, equipment (a so-called SimPC) is provided to those without an Internet connection. Once enrolled, panel members complete questionnaires on a monthly basis through the Internet. The recruitment of panel members is based on a random sample of addresses drawn from the community registers in co-operation with Statistics Netherlands.

In February 2009, a questionnaire on social integration and leisure time was administered to the LISS panel. A follow-up reminder was issued March 2009 for those who did not complete the survey in February. The questionnaire was administered to 8,160 panel members, and it was completed by 5,910 respondents (a response rate of 72.4 %).

The Internet penetration rate in the Netherlands was 86 % in 2009. Some older people, especially in the oldest age groups, might be more reluctant to participate in a web-based panel study because they have limited computer skills. Although the recruitment is not web-based and older people get special training in using the simple pc (SimPC), it could be that the oldest members participating in the panel might be somewhat better socially integrated than their counterparts in the general population since they can maintain social contact online while older people who don’t have access to the Internet cannot. There is no reason to assume, however, that this affects the representativeness of the sample.

### Measures

#### Social Connectedness

Social connectedness is an intangible construct and therefore hard to measure. Woolcock and Narayan (2000) state that one single, true measure for social connectedness is not possible because of the multidimensional character of the construct. Foley and Edwards (1999) agree that there is not just one correct way to measure social connectedness. By referring to the amount and quality of social relations in defining social connectedness, it is important to distinguish between the quantity and quality of older people’s social relationships. Bowling (1997) defines objective measures of social connectedness such as the number of social gatherings and the number of close relationships. Subjective measures involve satisfaction with social contacts, feelings of isolation or disconnectedness (Hughes et al. 2004), and loneliness (Russell et al. 1980). These subjective feelings reflect the difference between one’s desired and one’s actual relationships (Peplau and Perlman 1982).

##### *Number of Social Gatherings*

Number of social gatherings is measured by four questions: How often do you do the following?

- Spend an evening with family (other than members of your own household)
- Spend an evening with someone from your neighbourhood
- Spend an evening with friends outside your neighbourhood
- Visit a bar or café^2^

Answer categories: (1) never (2) about once a year (3) a number of times per year (4) about once a month (5) a few times per month (6) once or twice a week (7) almost every day. Answers to the four questions are added to form a single indicator with higher scores reflecting a greater degree of social connectedness through social gatherings.

##### *Number of Close Relationships*

Close relationships are those in which the participants’ lives are tightly interwoven, with both partners affecting and being affected in important ways (Hudson and Robins 1982). Examples include parents and their children, siblings, romantic partners, husbands and wives, and friends. To concentrate on the closest contacts, respondents were asked to name up to five persons with whom they discussed important things. As a result, the measure ranged from 0 to 5.

##### *Satisfaction with Social Contacts*

Respondents were asked about their satisfaction with social contacts,^3^ ranging from 0 (not at all satisfied) to 10 (completely satisfied).

##### *Feeling of Social Integration*

**Fig. 1:**
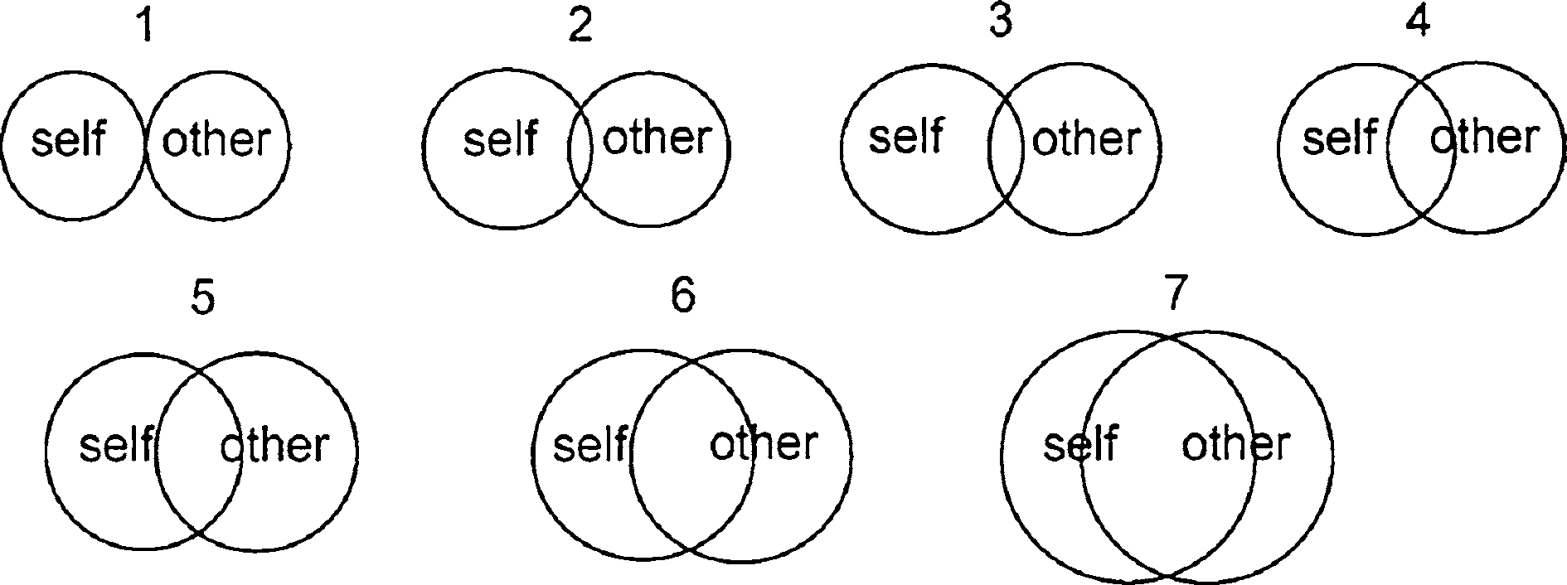
Feeling of social integration

A rating scale with circles was used to assess the degree to which people feel connected to other people (see Fig. 1). The measure was scored using the numbers shown on the figure, ranging from 1 to 7.

##### *Loneliness*

De Jong Gierveld and Van Tilburg (2006) define loneliness as an expression of negative feelings of missing relationships. They distinguish two components of loneliness. Emotional loneliness stems from the absence of an intimate relationship or a close emotional attachment. Social loneliness stems from the absence of a broader group of contacts or an engaging social network. The 6-item De Jong Gierveld Loneliness Scale has proven to be a reliable and valid measurement instrument for overall, emotional, and social loneliness and is suitable for large surveys (De Jong Gierveld and van Tilburg 2006).

Question: Can you indicate for each statement to what degree it applies to you, based on how you are feeling at present?

- I have a sense of emptiness around me
- There are enough people I can count on in case of a misfortune (reverse scale)
- I know a lot of people that I can fully rely on (reverse scale)
- There are enough people to whom I feel closely connected (reverse scale)
- I miss having people around me
- I often feel deserted

Answer categories: (1) no (2) more or less (3) yes

Answers are added to form an index with a theoretical range of 6–18, with higher scores indicating more feelings of loneliness.

#### Type of Social Contacts

Respondents could indicate the type of relationships with their closest contacts: partner, parent, brother or sister, child, colleague, club member, neighbor, friend. This resulted in eight binary variables, with one indicating that the respondent had the type of relationship and zero that he or she did not.

#### Leisure Activities

In selecting leisure activities it was tried to use the broadest array of activities given the range of activities included on the original survey. This resulted in the following ten leisure activities:

- *Voluntary work* over the past 12 months
- *Cultural activities* over the past 12 months (theatre, cabaret, concert of classical music, opera or operette, concert of popular music, dance event, ballet, cinema, film house, art gallery, museum)
- *Holiday* over the past 12 months (in the Netherlands or abroad)
- *Sports* on an average week
- Watching *TV* on an average week
- Listening to the *radio* over the past month
- Reading *books* over the past month
- Spending time on *hobbies* over the last 12 months (playing a musical instrument, singing, handicrafts, card games)
- *Shopping* over the last 12 months
- Using a *computer* on an average week

A binary variable was created for each activity, with one indicating that the respondent participated in the leisure activity and zero that he or she did not.

#### Age

Age was given in years and then classified into categories. Consistent with the literature (e.g. Bowling and Gabriel 2004; Hicks 1997) we compare younger adults (18–54 years), with the older adults in the age groups 55–64, 65–74, and 75+.

## Results

### Social Profile of Older Adults

The sample was divided into four age groups: 3,892 respondents fell in the age group 18–54, 1,171 in the age group 55–64, 637 in the age group 65–74 and 210 in the age group 75 and older. The total sample contained 2,713 men and 3,197 women (46 and 54 %, respectively). Table 1 presents the basic descriptive statistics for each of the key measures. In addition, Analyze of Variance tests (ANOVA) were conducted to identify whether any differences are evident among the age groups. Scheffe’s post hoc tests were conducted to identify homogeneous subsets for the variables of interest. The objective measures of social isolation—“social gatherings” and “number of close relationships”—decrease with age. The number of social gatherings shows a decrease from age 55. Respondents in the age group 18–54 report an average score on social gatherings of 15.43. This score decreases to 13.82 for the age group 55–64, 13.23 for the age group 65–23, and 12.68 for respondents aged 75 and older. The number of close relationships decreases from .62 to .63 for the age groups 18–54 and 55–64, respectively, to .51 for the age group 65–74 and .38 for respondents aged 75 and older. Subjective measures, “satisfaction with social contacts” and “feeling of social integration”, do not show a decrease with age. Respondents report higher rates for satisfaction with social contacts in older age groups, with the lowest rate for the age group 18–54 (7.18, 7.52, 7.76, and 7.52 for the groups 18–54, 55–64, 65–74, and 75+, respectively). It is notable that the same applies to feelings of social integration (4.66 for 18–54, and 5.06, 5.14, and 5.10 for the older age groups). Loneliness, on the other hand, increases with age. The oldest age group reported the highest score on loneliness (8.10, compared to 7.92, 7.70, and 7.86 for the age groups 18–54, 55–64, and 65–74, respectively). Although older people feel more connected to other people, this does not help them avoid experiencing feelings of loneliness. Loneliness and feelings of social integration seem to be two separate things.

**Table 1:** Mean score of key variables per age group

	Age Group				F
	18–54	55–64	65–74	75+	
N	3,892	1,171	637	210	
Social integration					
Social gatherings (4,…,28)	15.43^a^ (4.3)	13.82^b^ (3.6)	13.23^b,c^ (3.9)	12.68^c^ (4.1)	95.04**
Close contacts (0,…,5)	.62^a^ (1.2)	.63^a^ (1.2)	.51^a,b^ (1.1)	.38^b^ (.9)	4.14**
Satisfaction close contacts (0,…,10)	7.18^a^ (1.6)	7.52^b^ (1.6)	7.76^b^ (1.4)	7.52^b^ (1.8)	32.73**
Feeling of social integration (1,…,7)	4.66^a^ (1.5)	5.06^b^ (1.4)	5.14^b^ (1.4)	5.10^b^ (1.5)	34.55**
Loneliness scale (6,…,18)	7.92^a,b^ (2.5)	7.70^a^ (2.2)	7.86^a,b^ (2.1)	8.10^b^ (2.4)	3.29*
Leisure activity					
Voluntary work	.30^a^ (.5)	.46^b,c^ (.5)	.51^c^ (.5)	.41^b^ (.5)	64.45**
Cultural activities	.87^a^ (.3)	.82^a^ (.4)	.81^a^ (.4)	.74^b^ (.4)	15.14**
Holiday	.88^a^ (.3)	.84^a^ (.4)	.85^a^ (.4)	.71^b^ (.5)	21.41**
Sports	.59^a^ (.5)	.52^a,b^ (.5)	.48^b^ (.5)	.32^c^ (.5)	28.47**
TV	.96^a^ (.2)	.98^a^ (.1)	.98^a^ (.1)	.98^a^ (.2)	5.10*
Radio	.83^a^ (.4)	.85^a^ (.4)	.82^a,b^ (.4)	.77^b^ (.4)	3.16*
Books	.53^a^ (.5)	.54^a^ (5.)	.57^a^ (.5)	.58^a^ (.5)	1.96
Hobbies	.48^a^ (.5)	.54^a,b^ (.5)	.62^b^ (.5)	.54^a^ (.5)	16.90**
Computer	.97^a^ (.2)	.92^b^ (.3)	.87^c^ (.3)	.75^d^ (.4)	89.93**
Shopping	.75^a,b^ (.4)	.79^b^ (.4)	.79^b^ (.4)	.71^a^ (.5)	3.87**
Close contact					
Partner	.56^a^ (.5)	.46^b^ (.5)	.37^c^ (.5)	.27^d^ (.4)	10.19**
Parent	.39^a^ (.5)	.05^b^ (.2)	.00^b^ (.1)	.00^b^ (.2)	337.64**
Sibling	.28^a^ (.4)	.24^a,b^ (.4)	.19^b^ (.4)	.10^c^ (.3)	18.39**
Child	.09^a^ (.3)	.41^b^ (.5)	.49^c^ (.5)	.55^c^ (.5)	421.96**
Colleague	.16^a^ (.4)	.13^a^ (.3)	.04^b^ (.2)	.03^b^ (.2)	28.64**
Club	.04^a^ (.2)	.06^a,b^ (.2)	.08^b^ (.3)	.04^a^ (.2)	12.51**
Neighbor	.06^a^ (.2)	.09^a,b^ (.3)	.11^b^ (.3)	.10^b^ (.3)	12.75**
Friend	.61^a^ (.5)	.48^b^ (.5)	.39^b,c^ (.5)	.31^c^ (.4)	70.10**

Standard deviations between parentheses

Variables “voluntary work” to “friend” are binary variables (0 or 1)

^a,b,c,d^Homogeneous subsets for alpha =.05 with Scheffe’s post hoc test

* *p* < .05, ** *p* < .01

Table 1 shows, in addition to social connectedness variables, participation rates for the identified leisure activities. Participation rates for cultural activities, holiday, and sports are significantly lower for respondents in the age group 75+ compared to the other three age groups. For computer use the difference between each age group is significantly different from one another (97 % in the age group 18–54, 92 % in the age group 55–64, 87 % in the age group 65–74, and 75 % in the age group 75+). Listening to the radio decreases from the age of 75; participation rates drop down from around 83 % for respondents under the age of 75–77 % for respondents aged 75 and older. Participation in voluntary work increases with age from 30 % in the age group 18–54 to 46 % in group 55–64 to 51 % in group 65–74, but starts to decrease to 41 % in the age group 75 and older. Watching television is consistent across all age groups—96 % or more watch television. Reading books and having a hobby is consistent across age groups as well (around 54 % for both activities). Shopping activities increase with higher age groups, but decrease in the oldest age group (75+). This is probably due to a decrease in mobility.

Table 1 shows a decrease in social contacts with age. The frequency of having a partner or a sibling as a close contact decreases in higher age groups; in the age group 18–54 56 % reports to have a partner as a close contact compared to 27 % in the age group 75+; 28 % reports to have a sibling as a close contact in the age group 18–54 compared to 10 % in the age group 75+. Colleagues become less important for older respondents; the frequency with which colleagues are mentioned decreases from 16 % in the age group 18–54 to 3 % in the age group 75+. For obvious reasons, having a parent decreases from 39 % in the age group 18–54 to zero in the age group 75+. Discussing important matters with a child increases dramatically across age groups; 9 % of respondents in the age group 18–54 report having a child as a close contact compared to 55 % in the age group 75+. Having a club member as a close contact is stable across age groups (around 6 %), as is having a neighbor (around 9 %). While having a friend to discuss important matters to is most important for adults, a child as a close contact is the most important relationship for older people.

Taking all these results together, support for social disengagement theory is implied. Unfortunately this cannot be confirmed because of the cross-sectional nature of this study. Older people have less social gatherings and close contacts, and experience feelings of loneliness more often. On the other hand, older people are more satisfied with their social contacts, and even feel more connected to other people.

### Predicting Social Connectedness from Leisure Activities

In order to find out how participation in leisure activities predicts the five different measures of social connectedness, multiple regressions were carried out. Table 2 shows the results of the linear regressions for the objective measures of social integration (“number of social gatherings” and “close relationships”).

**Table 2:** Standard multiple regression on objective measures of social integration by leisure activities

Activity	Social gatherings				Number of close relationships			
	18–54	55–64	65–74	75+	18–54	55–64	65–74	75+
Voluntary work	−.024	.035	.087*	.002	.034	.015	.003	.52
Cultural activities	.078**	.077*	−.07	.070	.062**	.040	.075	.126
Holiday	.031	.044	.060	.042	.057**	.032	−.031	.069
Sports	.120**	.044	.090*	.090	−.001	.054	.155**	.084
TV	.007	.004	.020	.072	−.013	−.047	−.109**	.069
Radio	.005	.029	.032	−.023	−.013	.007	.035	−.007
Books	.005	.035	.051	.026	.074**	.072*	.088*	.091
Hobbies	.126**	.087**	.153**	.159*	.013	.024	.014	.158*
Computer	.022	.026	−.019	.008	.045**	.045	.013	.127
Shopping	.064**	.075**	.020	.072	−.032	−.045	.049	.038
N	3,892	1,171	637	210	3,892	1,171	637	210
R-squared	.058	.045	.055	.070	.023	.025	.066	.102
F	23.87**	5.41**	3.63**	1.49	9.01**	3.01**	4.44**	2.26*

Standardized beta coefficient presented

* *p* < .05, ** *p* < .01

Voluntary work, cultural activities, sports, hobbies, and shopping are significant predictors for the number of social gatherings a person has. For all age groups, hobbies—operationalized by playing a musical instrument, singing, handicrafts, and card games—is the best predictor for social gatherings. While reading books is not a successful predictor for social gatherings, it is one of the most important predictors for the number of close relationships an individual has. People who read apparently have more close contacts they can talk to about important matters. There may be another underlying construct influencing the relationship between reading books and the number of close relationships, for example education or habitus. In addition, going on holiday, cultural activities, and spending time behind the computer are significant indicators for the number of close relationships for people under the age of 55, but not for older people. Sport is positively associated with the number of close contacts for the age group 65–74. This could be because participation typically involves others. The passive leisure activities “TV” and “Radio” do not contribute to the number of social gatherings and close relationships. This confirms Putnam’s view (2000) that *doing* things accumulates more social contacts than *watching* or *listening* things. Doing things refers to productivity and involves action and creativeness and is often directed toward a (common) goal. More importantly, active leisure activities often involve cooperation. This could explain why active or productive activities generate social connectedness and passive or consumptive activities do not.

The subjective measures of social integration show a diverse range of predictors, as can be seen in Table 3. Satisfaction with social contacts is explained by sports, radio, reading books, hobbies, and shopping for the adult population (aged 18–54), with sports being the best predictor. Watching television reduces the satisfaction with social contacts for this age-group. Shopping is the most important leisure predictor for satisfaction with social contacts for people in the age group 55–64, hobbies for people in the age group 65–75, and cultural activities for people aged 75 and older. Shopping (for people under the age of 55) and voluntary work (for people aged 55–64) best predict feelings of social integration. No leisure activity has a significant positive effect on feeling of social integration for people aged 65 and older. The following activities contribute to a decrease in feelings of loneliness: voluntary work, cultural activities, holiday, sports, radio and reading books. Going on holiday is the most important indicator of reduced loneliness for people aged 64 and younger. Again, leisure does not contribute to feelings of loneliness for people aged 65–74. For the oldest age group (75+), cultural activities and sports reduce loneliness. There does not seem to be a general difference between objective and subjective measures of social connectedness in relation to leisure activities.

**Table 3:** Standard multiple regression on subjective measures of social integration by leisure activities

Activity	Satisfaction with social contacts				Feeling of social integration				Loneliness			
	18–54	55–64	65–74	75+	18–54	55–64	65–74	75+	18–54	55–64	65–74	75+
Voluntary work	−.010	.042	.058	−.033	.078**	.077*	.050	−.074	.012	−.061*	−.061	.069
Cultural activities	.026	.020	.098*	.295**	.024**	−.065*	.027	.139	−.057**	−.052	−.112	−.206*
Holiday	.027	.072*	.094*	−.016	.055	.007	.066	.020	−.124**	−.091**	−.165	.018
Sports	.106**	.075*	.027	−.002	.052**	.005	−.034	−.055	−.094**	−.067*	−.017	−.139*
TV	−.062**	−.020	.021	.049	−.013	.054	−.076	−.044	.014	−.018	−.022	−.106
Radio	.037*	.014	−.041	.138*	.040*	−.034	.062	.050	−.066**	.008	.002	−.028
Books	.054**	.058	.053	−.045	.043*	−.004	−.050	−.259**	−.060**	−.075*	−.055	.041
Hobbies	.037*	.005	.117**	.037	−.017	.069*	.076	.104	−.005	.028	−.064	−.064
Computer	−.005	.063*	−.051	−.044	−.020	−.005	−.049	.012	−.021	−.024	.100	.169*
Shopping	.040*	.085**	−.004	.043	.109**	.062*	.079	.097	−.015	−.015	−.010	.004
N	3,792	1,146	625	206	3,413	1,101	599	199	3,873	1,169	635	208
R-squared	.030	.041	.052	.110	.036	.020	.032	.090	.054	.040	.070	.091
F	11.54**	4.85**	3.39**	2.43**	12.72**	2.24*	1.95*	1.87*	22.13**	4.82**	4.72**	1.99*

Standardized beta coefficient presented

* *p* < .05, ** *p* < .01

The next section investigates which close contacts influence participation in leisure activities, in order to establish the people who could be the most successful facilitator for stimulating participation in the leisure sphere to increase social integration.

### Social Contacts Influencing Participation in Leisure Activities

In order to have an overview of the relationship between close social contacts and leisure, the three older age groups are combined. Table 4 shows probit estimations on leisure activities with social contacts for older adults aged 55 and older. Note that close contacts are measured by the people respondents name as social support in general (not related to a particular leisure activity). Therefore, the analyses do not reveal a real causal effect, but rather a correlation between type of social contact available and leisure activities.

**Table 4:** Probit estimation on leisure activities with social contacts for older people aged 55 and older

	Social Contacts							
	Partner	Parent	Sibling	Child	Colleague	Club member	Neighbor	Friend
Voluntary work	.12 (.1)	.72** (.3)	.22* (.1)	.18 (.1)	−.40* (.2)	.27 (.2)	.55** (.2)	.30** (.1)
Cultural activities	.43** (.1)	−.16 (.3)	.18 (.1)	.32** (.1)	.44 (.2)	.49 (.3)	.08 (.2)	.66** (.1)
Holiday	.74 (.1)	.15** (.4)	.11 (.2)	−.08 (.1)	.23 (.2)	.63* (.3)	−.06 (.2)	.54** (.1)
Sports	.38** (.1)	−.01 (.3)	.28* (.1)	−.09 (.1)	−.01 (.2)	.35 (.2)	.07 (.2)	.56** (.1)
TV	.19 (.1)	17.21 (.2)	.15 (.2)	.04 (.1)	−.67 (.4)	−.17 (.1)	.33 (.3)	.04 (.2)
Radio	.13 (.1)	.47 (.4)	.46** (.2)	.07 (.1)	.01 (.2)	−.26 (.2)	.23 (.2)	.01 (.1)
Books	.11 (.1)	.12 (.3)	.21 (.1)	.32** (.1)	.09 (.2)	−.07 (.2)	.12 (.2)	.67** (.1)
Hobbies	.39 (.5)	16.23 (.6)	1.55 (1.0)	.76 (.5)	.70 (1.0)	−.30 (.8)	.66 (1.0)	1.17* (.6)
Computer	.05 (.1)	.42 (.5)	.28 (.2)	.23 (.1)	1.21** (.4)	.60 (.4)	.09 (.3)	.82** (.2)
Shopping	−.12 (.1)	.26 (.3)	.14 (.1)	.41** (.1)	−.67** (.2)	−.16 (.2)	−.05 (.2)	.14 (.1)

B-coefficient reported. Standard error between parentheses

* *p* < .05, ** *p* < .01

Friends are positively correlated to all leisure activities except watching TV, listening to the radio, and shopping. Shopping is positively correlated to children as a type of close contact. Many social ties seem to be successful predictors of voluntary work: parents, siblings, and neighbours correlate to participation in voluntary work. Partners and children (core family) have a positive correlation with participation in cultural activities; going on holiday with parents and club members; sports with peers (partners and siblings); children with reading books; and colleagues with use of the computer. No social contacts other than friends positively correlate with participation in hobbies; hobbies are often organized around social groups outside the family context that consist of people who share a similar passion for a personal interest.

In all, hobbies were found to be the most important predictors of social gatherings, but only friends seem to stimulate participation in hobby activities. Reading books explained the number of close relationships significantly; children^4^ and friends can be used to stimulate reading for older adults. Cultural activities were the most important indicators of subjective measures of social integration for the oldest age group. Since this age group (aged 75 years and older) shows the most social isolation, this might be an important activity to target. The core family (partner and children) and friends can be used as stimulators for cultural participation.

## Discussion and Conclusion

This paper investigated how leisure activities can increase social integration of older adults. In addition, close contacts were taken into account to identify which people may serve as successful stimulators of participation in leisure activities to help reduce social isolation of older adults.

Older people have less social contacts and participate in less leisure activities. They are more satisfied with their social contacts and feel more connected to others than younger adults, however. This indicates that loneliness and social connectedness are two separate things. Active leisure activities contribute more to social integration than passive activities (see Putnam 2000). All identified leisure activities have a significant influence for the adult population (<55 years of age). Fewer leisure activities have a significant effect on the older population. Cultural activities, reading books, and hobbies have the strongest effect for people aged 55 and older. These activities can be characterized as “serious leisure” (see Kleiber 1999). Serious leisure is the systematic pursuit of a leisure activity that is sufficiently substantial and interesting for the participant to find a career there in the acquisition and expression of its special skills and knowledge. Relating this to Putnam (2000), serious leisure activities may be the ultimate form of productive activities and hence, the best predictor of social integration. It is important to note that the significance of hobbies in explaining social integration is difficult to assess. In this study, only four hobbies were taken into account (playing a musical instrument, singing, handicrafts, and card games). One can assume that more types of hobbies people participate in increase the effect of hobbies in relation to other leisure activities. Participation in hobbies might be the most important leisure activity for predicting social integration. Further research may clarify this issue.

The ten leisure activities used in the various regression models explained between 2 and 11 % of the variance in social connectedness variables. Given all of the possible social, economic, and situational factors that could play a part in determining the level of social connectedness, the amount of variance explained by just a few leisure activities is remarkable. Stimulating participation in leisure activities may therefore be an effective tool in reducing social isolation among older adults. This could help reduce bad physical and mental health associated with social isolation (Findlay 2003; Pettigrew 2007), thereby reducing medical costs. Public policy debates need to focus on social issues as well as physical and mental issues in order to increase quality of life of older adults.

A specific strategy to increase social integration for older people could be facilitating their connections with others via leisure activities. Local communities can seek to increase social support through public announcements, advertisements, peer- family- or multi-generation programs at reduced rates, or special events that may bring together older people and their close social contacts and hence provide a means to develop social connectedness. In addition, public policies can develop special programs to select, train, and stimulate the close contacts that this study has shown contribute to leisure participation and social integration, thereby improving quality of life for older adults. Selection, training, and support of facilitators to stimulate social interaction among older people appear to be important factors underpinning successful interventions (Findlay 2003). This of course in addition to improving societal structures that provide opportunities that would facilitate social interaction.

As mentioned by Pettigrew (2007), older people might be averse to seeking the company of those of their own age; programs that facilitate intergenerational contact may be more effective in alleviating loneliness. This study shows for each leisure activity which kind of contact (partner, child, friend, etc.) could be most appropriate. In addition, this approach also shows which kind of contact could serve as a communication audience, which may be more effective than attempting to encourage the older person to take the initiative in arranging interactions.

The results show that neighbors do not seem to play a role in stimulating older people to participate in leisure activities. Friends serve most often as stimulators. Partners play a role in participation in cultural activities and sports; parents stimulate participation in voluntary work and holidays; siblings facilitate voluntary work and sports; and children participation in cultural activities, reading books, and shopping. The oldest age group is the most socially isolated group and cultural activities were found to be the most important indicators of subjective measures of social integration for this group. This seems the most important target group in terms of improving quality of life. Partners, children, and friends can could serve as facilitators for cultural participation for the oldest age-group (75 years and older).

As mentioned earlier, social connectedness is a difficult concept to define, measure, and use. This study shows that leisure activities relate to both objective and subjective measures of social connectedness. Subjective measures may not be showing the same decline in social connectedness along age groups as objective measures do. In addition, the different subjective measures identified in this study do not seem to be measuring the same thing. “Satisfaction with social contacts” and “feeling of social integration” exhibit many similarities, but seem to differ from the loneliness scale used. Loneliness and feelings of social connectedness seem to be two separate things, and although people could be satisfied with their social contacts, they still could feel lonely. There does not seem to be a big difference in the relationship between leisure and objective measures on the one hand, and leisure and subjective measures on the other; most leisure activities relate to both types of measures.

The use of a cross-sectional research design was chosen because of lack of longitudinal studies available, but was not optimal. To develop a theoretical model for quality of life in old age, longitudinal research is needed to measure any dynamic features, cohort, and ageing effects. Future research could best survey older people over time, to distinguish between age and cohort effects. Unfortunately, due to the cross-sectional design, no real causal relationships could be determined within this study. Future research to confirm these results is therefore warranted. With leisure participation being measured as simply “yes” or “no”, the intensity of engagement is not reflected within this study. Consequently, casual participation (i.e., once or twice in the past 12 months) is treated the same as regular participation (i.e., every day). This has to be taken into account when interpreting the results. For example, hobbies, which are typically undertaken regularly, are the most important predictor of the number of social gatherings. On the other hand, going on holiday, typically done irregularly, did not show a relation with the number of social gatherings, but exhibited a significant relationship with subjective measures of social connectedness. Leisure diversity was also not taken into account. It could well be that the more activities reported, the more diverse a person’s leisure repertoire and therefore possible more opportunities for social interactions. In all, the relationship between leisure activities and social integration needs to be further explored in order to get a better understanding of how leisure activities can stimulate social integration.

## Footnotes

1 This is somewhat in contrast to the civic approach to social capital of Putnam (1995; 2000), who refers to networks, norms and trust.

2 The question was not specific to engaging alone or with others.

3 Satisfaction with social contacts was not defined for respondents.

4 Most of the literature suggests that parental role modeling results in children adopting similar behaviors. So, reading may be associated because the children are engaging in an activity introduced to them by parents (i.e., older adults).
